# Prognosis-related genes participate in immunotherapy of renal clear cell carcinoma possibly by targeting dendritic cells

**DOI:** 10.3389/fcell.2022.892616

**Published:** 2022-09-29

**Authors:** Guodong Fang, Xudan Wang

**Affiliations:** ^1^ Department of Pathology, Shaanxi Provincial People’s Hospital, Xi’an, China; ^2^ Department of Hepatobiliary Surgery, Xijing Hospital, Fourth Military Medical University, Xi’an, China

**Keywords:** immunotherapy, prognosis, risk score, dendritic cell, clear cell renal cell carcinoma

## Abstract

Tumor immunotherapy has become one of the most promising approaches to tumor treatment. This study aimed to screen genes involved in the response of clear cell renal cell carcinoma (ccRCC) to immunotherapy and analyze their function. Based on the Gene Expression Omnibus and The Cancer Genome Atlas datasets, we screened out nine differentially expressed genes (TYROBP, APOC1, CSTA, LY96, LAPTM5, CD300A, ALOX5, C1QA, and C1QB) associated with clinical traits and prognosis. A risk signature constructed by these nine genes could predict the survival probability for patients at 1 year, 3 years, and 5 years. The immune checkpoint blockade response rate in the high-risk group was significantly higher than in the low-risk group (49.25% vs. 24.72%, *p* ≤ 0.001). The nine prognosis-related genes were negatively correlated with activated dendritic cells in the low-risk group but not in the high-risk group. qRT-PCR, immunohistochemistry, and immunofluorescence showed that the nine prognosis-related genes were associated with dendritic cell activity and the PD-1 positive staining rate. In conclusion, the nine prognosis-related genes have a high prognostic value. The patients in the high-risk group were more likely to benefit from immunotherapy, and the mechanism might be related to the release of dendritic cell-mediated immunosuppression.

## Introduction

Clear cell renal cell carcinoma (ccRCC) is a malignant tumor of the urinary system, accounting for approximately 75% of renal cancers (Deleuze et al., 2020). Immunotherapy drugs for ccRCC have made great progress, from the initial use of interleukin and interferon to the emergence of immune checkpoint inhibitors (ICIs) (Brown et al., 2020; Motzer et al., 2020). An immune checkpoint (IC) is an inhibitory pathway of the immune system, and its main function is to prevent the immune system from harming normal cells during a state of over-activation (Li et al., 2019). ICs are closely related to the immune escape of tumors, and inhibition of key molecules of ICs has become the main approach to tumor immunotherapy (Petitprez et al., 2020).

Common targeted IC molecules include programmed death receptor-1 (PD-1)/programmed cell death 1-ligand 1 (PD-L1), and cytotoxic T lymphocytes (CTLA4). In addition, mismatch repair (MMR)/microsatellite instability (MSI) and TMB are also used for tumor immunotherapy evaluation (Zhu et al., 2018). The expression levels of these indicators are very different in different tumors, which seriously restricts the application of immunotherapy. A study of the positive rates of PD-L1, TMB, and MSI in 155 kidney cancer samples found that the positive rates of TMB and MSI were only 1/155 each, while the positive rate of PD-L1 was higher (46/155), and 108/155 tumors were negative for all three (Vanderwalde et al., 2018). Another study involving 244 patients with renal transitional cell carcinoma found that high TMB (>20 mutations/Mb) occurred in only 6.3% of cases (Chalmers et al., 2017). Therefore, lack of molecular markers that can effectively predict treatment outcomes remains a major challenge for clinicians.

TIDE scores, a new scoring system that integrates T-cell dysfunction and cell rejection markers to simulate tumor immune escape, can predict ICB response rates and have been shown to be superior to PD-L1 and TMB in predicting the immunotherapy responses of malignant melanoma (Jiang et al., 2018). However, there are no clinical studies of TIDE in ccRCC to date. The emergence of a new generation of genetic detection technology opens the door to big data research, and various new molecular markers are constantly emerging. A clinical study conducted genetic testing on 212 high-risk ccRCC samples. Sixteen prognostic genes were selected and used to construct a recurrence risk signature for ccRCC, and the efficacy of sunitinib was predicted and evaluated for tumors with different risk levels. The potential application of this study is related to FDA approval of sunitinib as an adjunct therapy for patients at high risk of kidney cancer recurrence (Rini et al., 2018).

In this study, the transcriptome data and clinical data of ccRCC were downloaded from the GEO and TCGA databases, the differentially expressed genes (DEGs) related to prognosis were screened, the risk signature was constructed, and the risk score was calculated. Then, we further explored the diagnostic and prognostic values of the risk score and its role in immunotherapy, aiming to provide new predictive methods and therapeutic targets for immunotherapy of tumors.

## Data and methods

### Dataset acquisition

Three gene microarrays with ccRCC (GSE14994, GSE15641, and GSE53757) were obtained from the Gene Expression Omnibus (GEO) dataset (https://www.ncbi.nlm.nih.gov/gds/). GSE14994 included 90 ccRCC samples and 48 normal renal cortex samples, GSE15641 included 32 ccRCC samples and 23 normal kidney samples, and GSE53757 included 72 ccRCC samples and 72 matched normal kidney samples. In addition, the transcriptome data and clinical information of ccRCC were downloaded from The Cancer Genome Atlas (TCGA) datasets, including 534 tumor samples and 72 normal samples.

### Differentially expressed gene (DEG) analysis

GEO2R was used to analyze the DEGs of the three gene microarrays based on a threshold value with |log_2_ fold change (FC)| > 1 and *p* value <0.05. The DEGs were visualized by the ggplot2 package (v3.3.3) in R. The intersection genes of each dataset were analyzed using the R package “Venn”.

### Weighted gene co-expression network analysis (WGCNA)

WGCNA was performed with the R package “WGCNA” (v1.69) on tumor samples from the TCGA dataset. The clinical traits such as age, sex, stage, and grade were included in the WGCNA to screen the module that was most closely related to these traits. Then, the expression of the genes assigned to the module was visualized by the ggplot2 package, comparing tumor samples and normal samples from the TCGA datasets.

### Univariate and multivariate Cox regression analyses

Kaplan–Meier (K-M) Plotter (http://kmplot.com/analysis/) was applied to screen the genes related to the overall survival (OS) of ccRCC, excluding genes with *p* value >0.05 and obtaining nine prognosis-related genes. Then, the 533 ccRCC samples were randomly divided into the training set (n = 373) and the verification set (n = 160) at a ratio of 7:3. The prognosis-related genes were included in the univariate and multivariate Cox regression analyses by using the R package “survival” in the training set to obtain the regression coefficient of each gene. To avoid missing some important factors, we did not perform screening at this step. The nine genes were used to construct the risk signature, and risk scores were calculated by using the linear model and predicting the coxph function based on the gene expression values after regression coefficient weighting (Liu et al., 2019). The risk score formula was as follows:

$$Risk\;score=\sum_{i=1}^{N}Expi\times \beta i,$$

where Exp is the expression value of each gene in the sample and β : is the regression coefficient of the multivariate Cox regression analysis for each gene.

On the basis of this formula, the risk scores of each patient from the training set and verification set were calculated. Then, according to the median risk score, the samples were divided into high-risk and low-risk groups in both the training and verification sets. The K–M curve and receiver operating characteristic (ROC) curve were visualized by using the survminer package and survivalROC package, respectively, in R.

In addition, the risk score and clinical traits, including age, sex, stage, and grade, were included in the multivariate Cox regression analysis to evaluate the independent prognostic factors with a threshold value of *p* value <0.05. The nomogram (Cox) was constructed by using the rms package (v5.1-4) in R to predict the patient’s survival probability at 1, 3, and 5 years. The consistency of the nomogram (Cox) was assessed by calibration curves. The nomogram (logistic) was constructed by a logistic regression model in R to predict the patient’s risk of death.

### Functional enrichment analysis

Functional enrichment analysis (GO-BP) of the nine prognosis-related genes was performed and is shown by the ClueGO plug-in of Cytoscape software (v3.7.2).

### Immunotherapy evaluation

Mutation data of ccRCC patients were downloaded from the TCGA datasets, the values of the tumor mutation burden (TMB) of each sample were calculated by using the R package “TCGAmutations,” and the results were visualized by using the “ggplot2” package.

According to the transcriptome data of ccRCC patients from the TCGA datasets, the Tumor Immune Dysfunction and Exclusion (TIDE) of each sample was analyzed by the TIDE algorithm (http://tide.dfci.harvard.edu/) (Jiang et al., 2018) and visualized by the “ggplot2” package.

The immunophenoscore (IPS) is an immunotherapy evaluation index that only uses the expression of PD-L1 in tumor-related immune cells (lymphocytes, macrophages, etc.) as an evaluation index to distinguish benefiting cohorts (Liu et al., 2020). The IPS data of ccRCC patients were downloaded from The Cancer Immunome Atlas (TCIA, https://tcia.at/home) datasets and visualized by the “ggplot2” package.

### Immune infiltration analysis

To further explore the mechanisms of action of the prognosis-related genes, the ESTIMATE algorithm (https://bioinformatics.mdanderson.org/estimate/) was employed to analyze the relationship between the risk score and stromal and immune cells. The tumor-infiltrating immune cell fraction of each ccRCC and matched normal sample was calculated by the CIBERSORT algorithm (https://cibersort.stanford.edu/index.php) and visualized by the “ggplot2” package. Excluding the immune cells that were not significantly different between the ccRCC samples and normal samples, the correlations of the prognosis-related genes and the differential immune cells were calculated by Pearson’s correlation analysis and visualized by the R package “ggcorrplot”. The molecular markers of the activated dendritic cells were downloaded from the TCIA datasets, and the correlation of the expression patterns of the markers and the prognosis-related genes was calculated by Pearson’s correlation analysis and visualized by Cytoscape software.

### Real-time fluorescence quantitative PCR

Paraffin tissue blocks and clinical data from 22 ccRCC patients were collected from Shaanxi Provincial People’s Hospital. RT-PCR was employed to detect the expression of the prognosis-related genes and activated dendritic cell markers. A DNA/RNA extraction kit (AmoyDx, Xiamen, China) was used to extract RNA from the tissue paraffin blocks. The Evo M-MLV RT kit and SYBR^®^ Green Premix Pro Taq HS kit (Accurate Biology, Changsha, China) were used to conduct two-step fluorescence quantitative PCR. The Cq value was employed to calculate the gene expression based on 2^−ΔΔCt^. The internal reference gene was GAPDH.

### Immunohistochemistry

Paraffin slices were produced from tissue paraffin blocks and dewaxed and dehydrated. Then, we used H_2_O_2_ to block endogenous peroxides and conducted antigenic thermal repair. The slices were blocked with fetal bovine serum (FBS) at 37°C for 30 min, incubated with the primary antibody (PD-1 and PD-L1 antibodies, Abcam, United Kingdom) at 4°C overnight, and then incubated with the secondary antibody at 37°C for 1 h. DAB-H_2_O_2_ was dropped onto the slices to color the sites of the antibody binding for approximately 10 min. The slices were counterstained with hematoxylin, and then the conventional slide-sealing process was conducted. Images were collected with an optical microscope (Olympus, Japan), with 400× magnification. The positive cell ratio, H-score, and immunohistochemical staining results (IRS) were analyzed by three pathologists with intermediate professional titles.

### Immunofluorescence

The aforementioned slices were blocked with FBS and incubated with a fluorescently labeled primary antibody (PD-1 and CD11c antibodies, Abcam, United Kingdom) at 4°C overnight and then incubated with a fluorescently labeled secondary antibody at room temperature for 1 h in the dark. DAPI was dropped onto the slices to color the sites of antibody binding for approximately 10 min, and then the conventional slide-sealing process was conducted. Images were collected with a fluorescence microscope (Olympus, Japan), 1,000×.

### Statistical analysis

All statistics were analyzed by R software (v4.1.0). The statistical significance of the continuous variables was analyzed by the Wilcoxon rank-sum test. The correlation was analyzed by Pearson’s correlation analysis. A two-sided *p* value <0.05 was considered statistically significant.

## Results

### Screening of the DEGs in ccRCC

**FIGURE 1:**
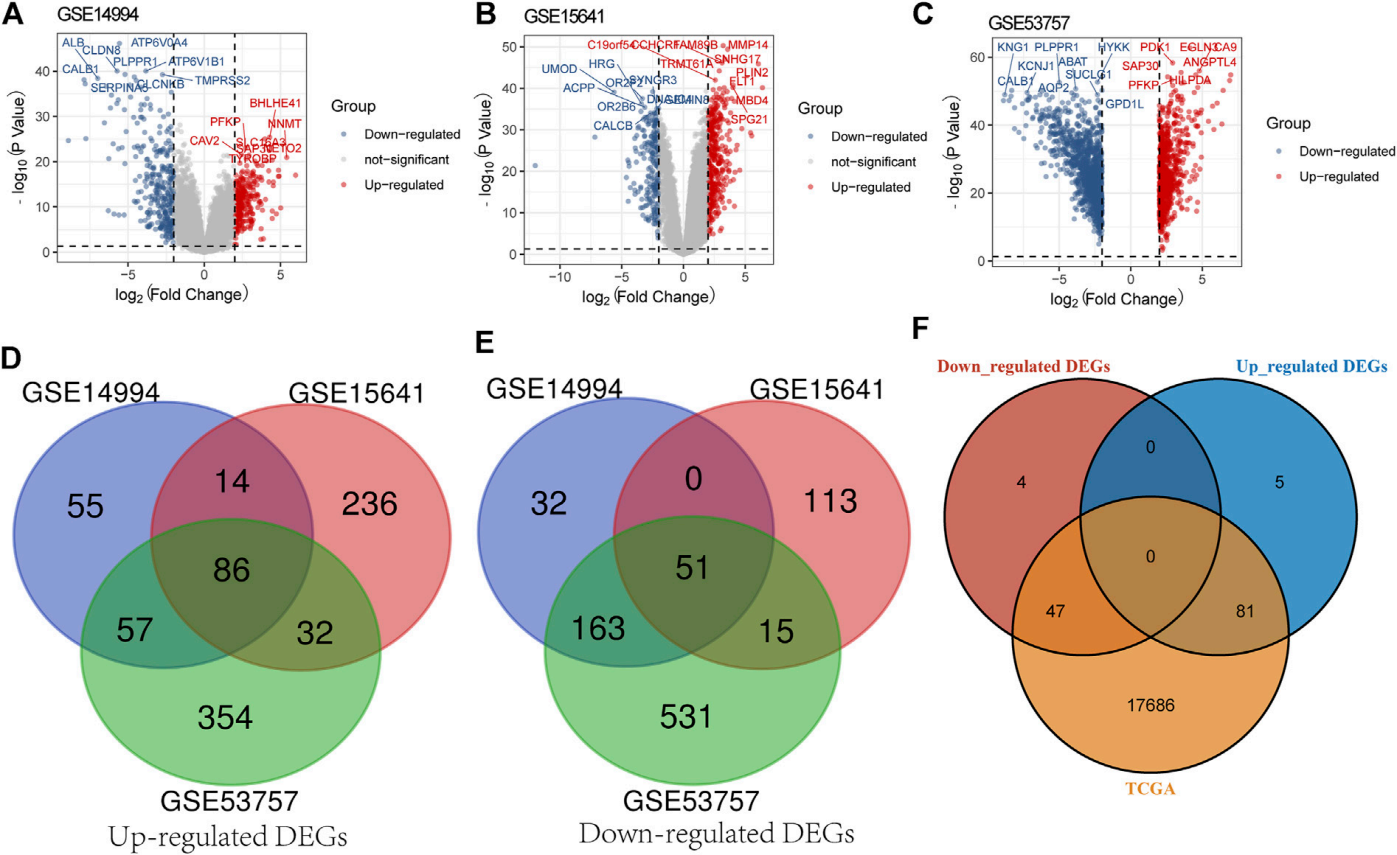
Screening for DEGs. **(A)** Screening for DEGs of GSE14994. **(B)** Screening for DEGs of GSE15641. **(C)** Screening for DEGs of GSE53757. **(D)** Venn diagram presents common upregulated DEGs for the three gene microarrays. **(E)** Venn diagram presents common downregulated DEGs for the three gene microarrays. **(F)** Venn diagram presents the downregulated and upregulated DEGs common to the three gene microarrays and the TCGA database.

According to a threshold value with |log_2_ FC| > 1 and *p* value <0.05, we obtained 458, 547, and 1,289 DEGs between the ccRCC samples and normal samples from GSE14994 (Figure 1A), GSE15641 (Figure 1B), and GSE53757 (Figure 1C), respectively. Then, we used a Venn diagram to intersect the downregulated DEGs and upregulated DEGs of the three gene microarrays, obtaining 86 common upregulated DEGs (Figure 1D) and 51 common downregulated DEGs (Figure 1E). Furthermore, we intersected these genes with those in the TCGA database, obtaining 47 downregulated DEGs and 81 upregulated DEGs (Figure 1F). These 128 DEGs were shared by the three gene microarrays and the TCGA database.

### Identification of clinical trait-related genes

**FIGURE 2:**
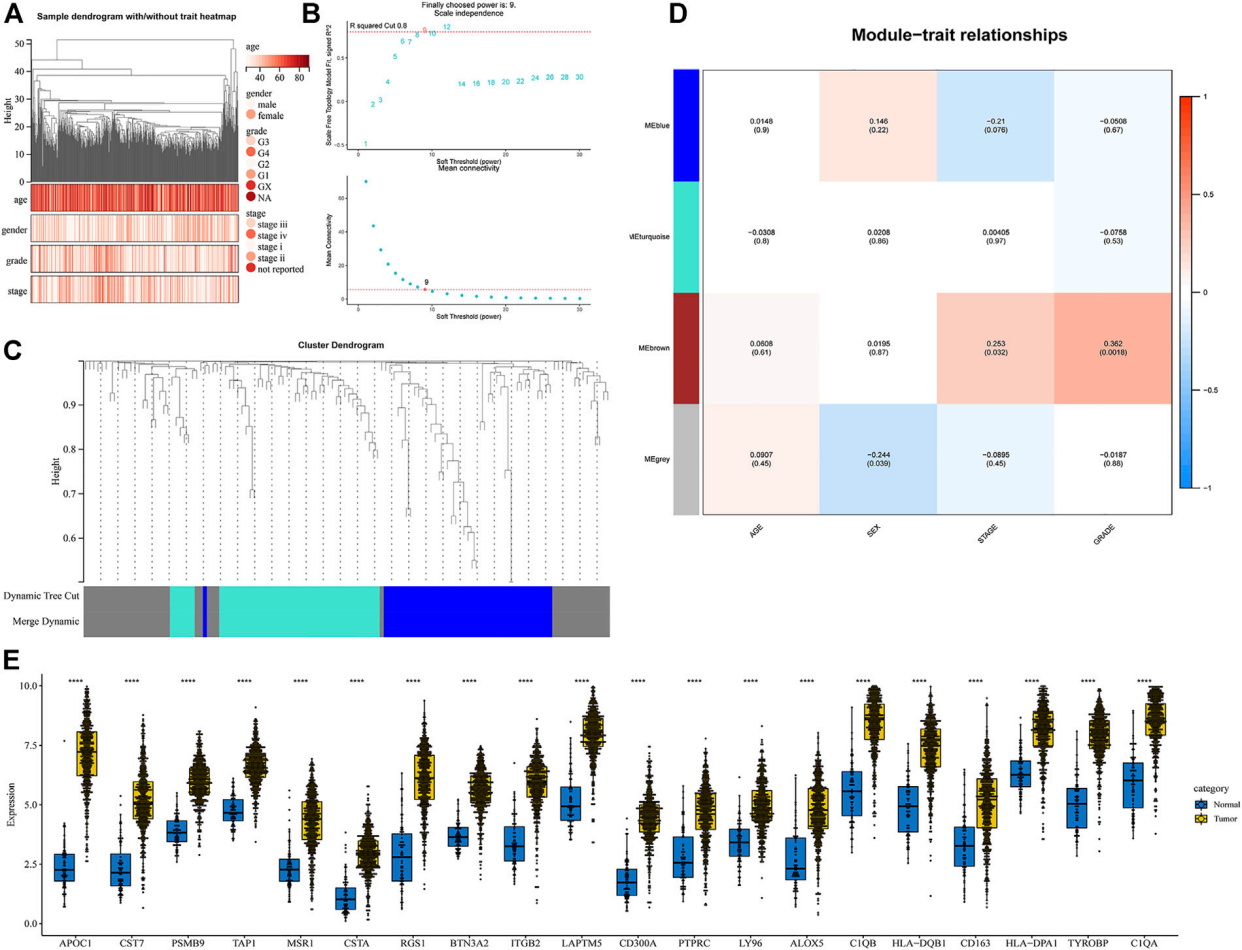
Identification of the clinical trait-related genes. **(A)** Clustering dendrogram of all samples. **(B)** Analyses of the scale-free index and mean connectivity for multiple soft-threshold powers. **(C)** Cluster dendrogram of all genes. **(D)** Correlation heatmap of the module eigengenes and clinical traits. **(E)** Expression of the 20 clinical trait-related genes based on the TCGA database.

A gene co-expression network of the aforementioned 128 genes was constructed by the R package “WGCNA” to identify clinical trait-related genes. After excluding samples with incomplete clinical traits, the 533 samples from the TCGA database were included in the gene co-expression network. A sample clustering tree is shown in Figure 2A. In this study, we chose the soft-threshold *β* = 9 (scale free *R* ^2^ = 0.8) to construct a scale-free network (Figure 2B). The four modules were identified according to dynamic tree clipping and average hierarchical clustering (Figure 2C). Pearson’s correlation analysis of the modules and the clinical traits suggested that the brown module was the most closely related to clinical stage and grade (Figure 2D), so this module was considered a clinical trait-related module, which included 20 genes. These genes were all upregulated in the ccRCC tissue samples from the TCGA database (Figure 2E). The genes included in each module are presented in Supplementary Table S1.

### Identification of hub prognosis-related genes

Kaplan–Meier survival analysis was performed to identify the genes most closely related to OS in ccRCC. According to a threshold value of *p* < 0.05, we obtained nine prognosis-related genes: ALOX5, APOC1, C1QA, C1QB, CD300A, CSTA, LAPTM5, LY96, and TYROBP (Table 1).

**TABLE 1:** Kaplan–Meier survival analysis of 20 clinical trait-related genes.

Gene	Low-expression cohort (months)	High-expression cohort (months)	HR (95% CI)	P value
ALOX5	70.17	31.07	2.27 (1.50–3.43)	**5.9e-5**
APOC1	52.97	28.17	1.61 (1.19–2.18)	**0.0019**
BTN3A2	85.47	120.50	0.86 (0.63–1.16)	0.3108
C1QA	118.47	66.00	1.77 (1.30–2.40)	**0.0002**
C1QB	118.47	73.00	1.58 (1.17–2.13)	**0.0024**
CD163	118.47	75.20	1.19 (0.86–1.66)	0.292
CD300A	118.47	76.63	1.38 (1.01–1.88)	**0.0427**
CST7	44.77	31.07	1.33 (0.98–1.79)	0.0628
CSTA	118.47	70.17	1.56 (1.14–2.13)	**0.0055**
HLA-DPA1	75.20	118.47	0.74 (0.53–1.02)	0.0682
HLA-DPB1	80.63	120.50	0.76 (0.56–1.02)	0.0681
ITGB2	118.47	78.10	1.28 (0.95–1.73)	0.1051
LAPTM5	118.47	73.00	1.49 (1.10–2.01)	**0.0088**
LY96	118.47	55.37	2.04 (1.51–2.77)	**2.5e-6**
MSR1	80.63	92.13	0.82 (0.59–1.14)	0.2388
PSMB9	92.13	79.53	1.23 (0.89–1.69)	0.2069
PTPRC	71.50	118.47	0.74 (0.54–1.01)	0.0568
RGS1	48.77	36.40	1.44 (0.99–2.09)	0.0558
TAP1	92.13	79.53	1.22 (0.89–1.68)	0.2201
TYROBP	45.93	27.30	1.66 (1.23–2.24)	**0.0009**

Note: The bold type represents a *p* value <0.05.

### Construction of the risk signature

**FIGURE 3:**
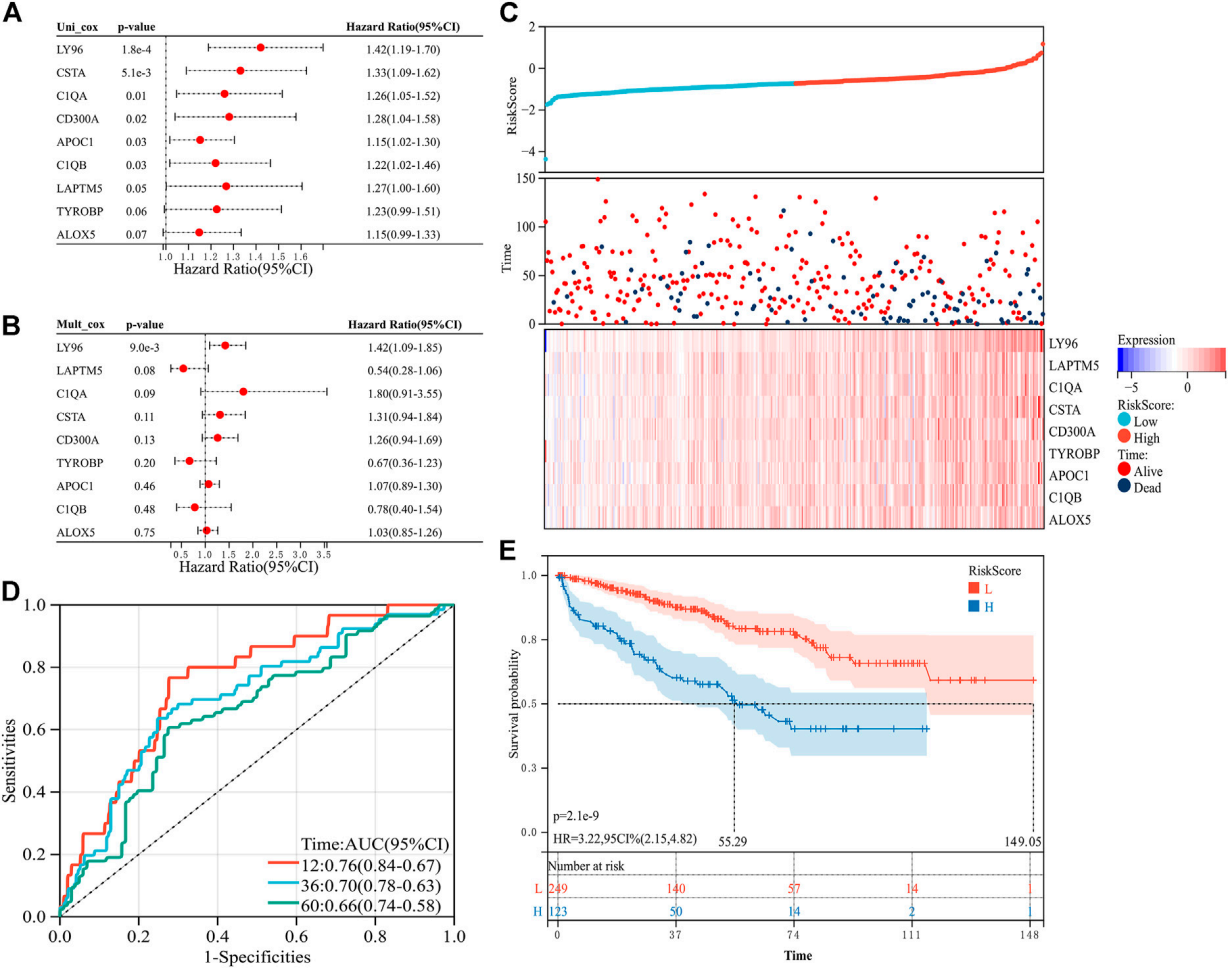
Construction of the risk signature with nine prognosis-related genes in the training set. **(A)** Univariate Cox regression analysis. **(B)** Multivariate Cox regression analysis. **(C)** Distribution of the risk score and survival of patients and the expression pattern of the nine prognosis-related genes in the high- and low-risk groups. **(D)** Receiver operating characteristic (ROC) curves of overall survival (OS) at 1 year, 3 years, and 5 years **(E)** K–M survival curve of the OS in the high- and low-risk groups.

**FIGURE 4:**
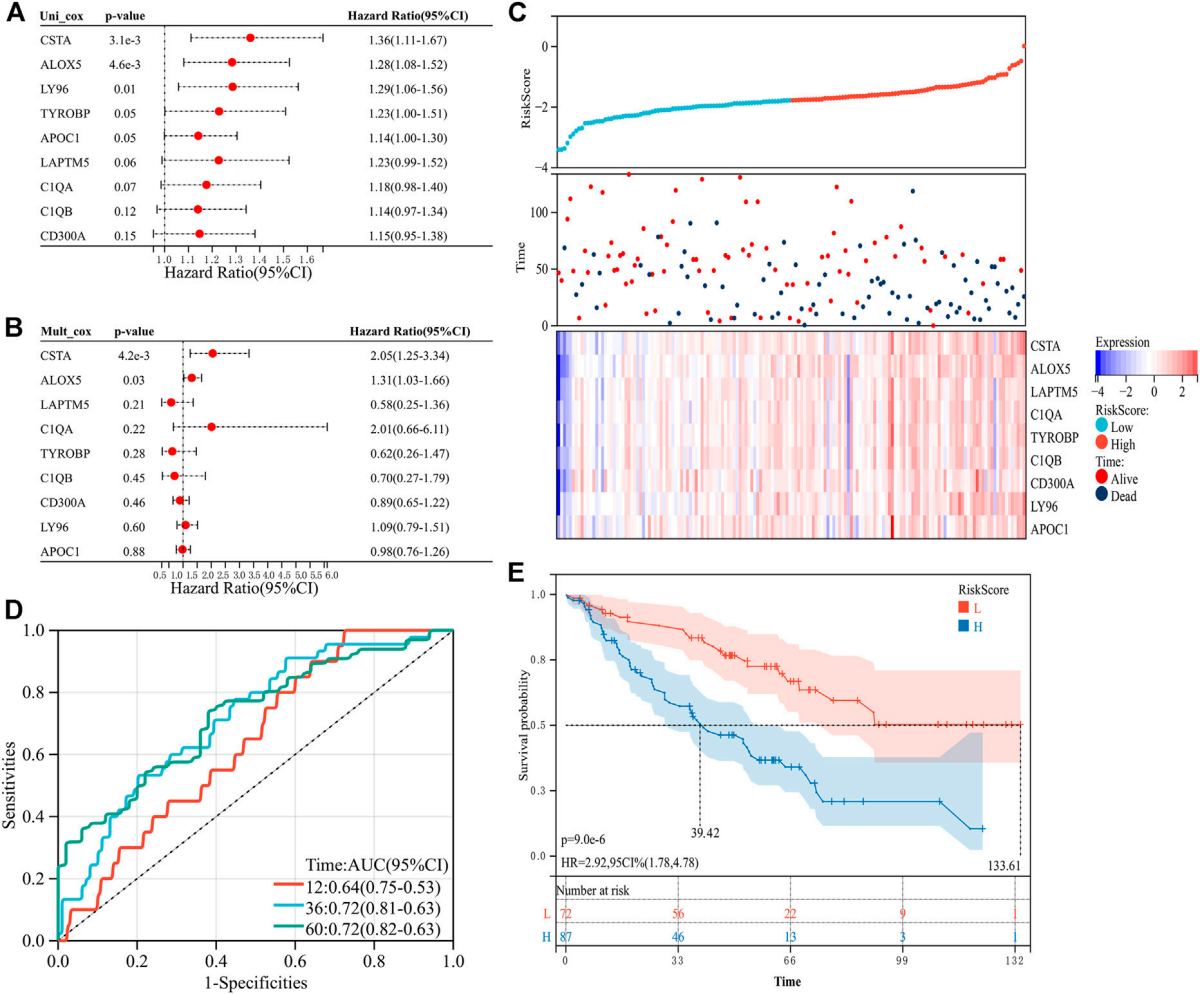
Construction of the risk signature with nine prognosis-related genes in the verification set. **(A)** Univariate Cox regression analysis. **(B)** Multivariate Cox regression analysis. **(C)** Distribution of the risk score and survival of patients and the expression pattern of the nine prognosis-related genes in the high- and low-risk groups. **(D)** ROC curves of OS at 1 year, 3 years, and 5 years. **(E)** K–M survival curve of the OS in the high- and low-risk groups.

To explore the predictive value of the nine prognosis-related genes, the 533 samples were randomly divided into the training set (n = 373) and the verification set (n = 160) at a ratio of 7:3. In the training set, the prognosis-related genes were included in the univariate and multivariate Cox regression analyses (Figures 3A, B). To avoid missing some important factors, this step did not exclude genes to obtain the regression coefficient of each gene. Then, according to the regression coefficient and expression data of each gene, we calculated the risk score of each sample (Supplementary Table S2). Based on the median value of the risk score, the samples in the training set were divided into a high-risk group and a low-risk group. We found that the number of deaths in the high-risk group was higher than that in the low-risk group, and the nine prognosis-related genes were all highly expressed in the high-risk group (Figure 3C). In addition, the risk score had higher accuracy for the survival prediction of ccRCC patients (AUC_1-year_ = 0.76, AUC_3-year_ = 0.70, and AUC_5-year_ = 0.66, Figure 3D). The patients in the high-risk group had a worse prognosis than those in the low-risk group (*p* = 2.1e-9, HR = 3.22, 95% CI = 2.15-4.82, Figure 3E). In addition, similar results were obtained in the validation set (Figures 4A–E, Supplementary Table S3). These results suggested that the risk signature constructed from the nine prognosis-related genes had better value for prognostication.

### Relationship between the risk score and clinical traits

**FIGURE 5:**
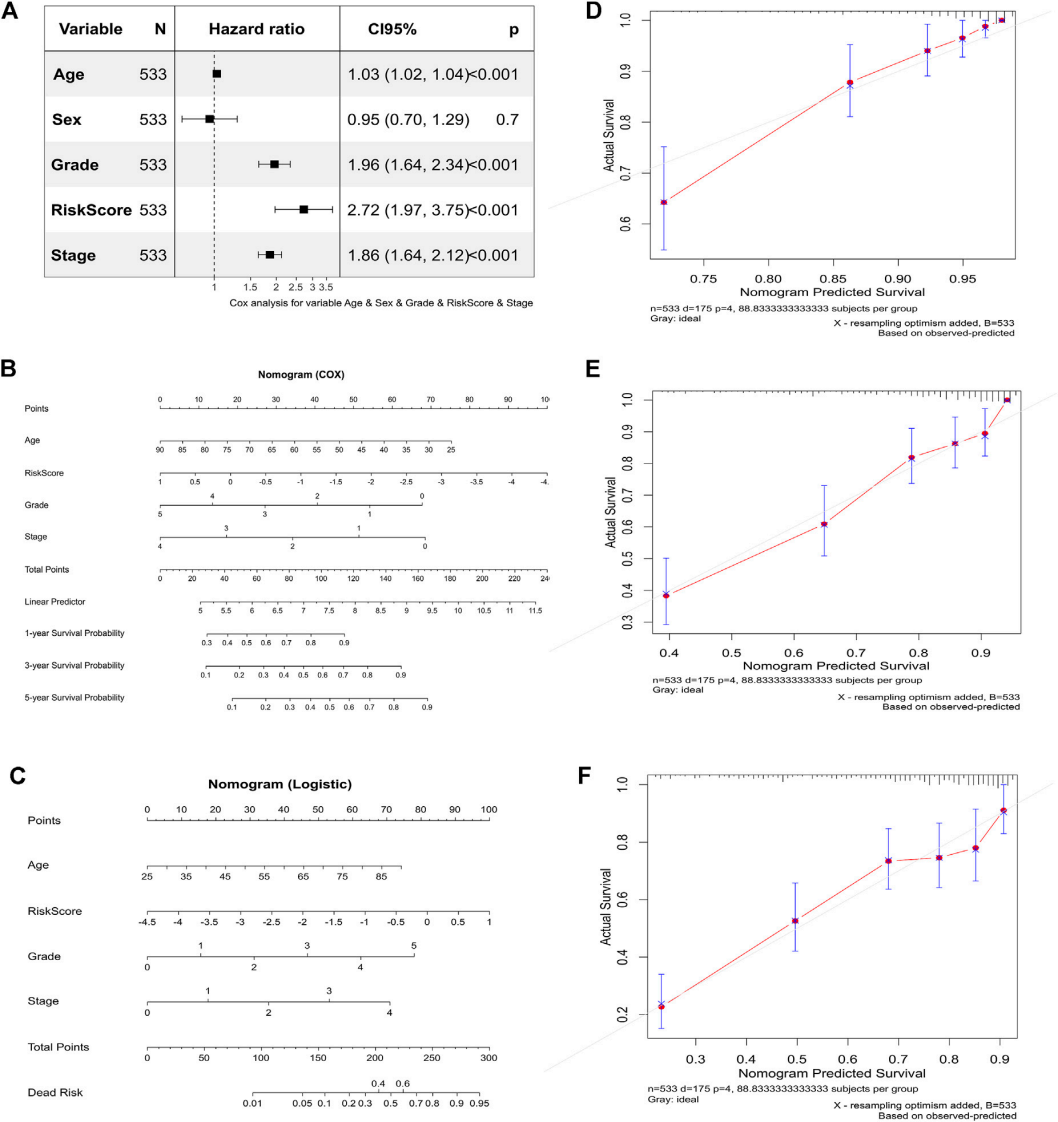
Correlation between the risk score and clinical traits and their predictive value. **(A)** Multivariate Cox regression analysis for predicting independent prognostic factors. **(B)** Nomogram based on the Cox regression analysis. **(C)** Nomogram based on the logistic regression analysis. **(D)** The calibration curve for 1 year. **(E)** Calibration curve for 3 years. **(F)** Calibration curve for 5 years.

We employed multivariate Cox regression analysis to investigate the relationship between the risk score and clinical traits. Age, sex, stage, and grade were included in the analysis. We found that age, stage, grade, and risk score were significantly associated with an unfavorable prognosis of ccRCC patients (*p* < 0.001, Figure 5A), suggesting that the risk score might be useful as an independent prognostic factor for ccRCC patients. Then, we used these significant factors to construct nomograms based on the Cox regression analysis or logistic regression analysis. The greater the value of these four factors, the lower the probability of survival of the patients at 1, 3, and 5 years (Figure 5B). Similarly, the greater the value of these four factors, the greater the risk of death (Figure 5C). A calibration curve was used to evaluate the consistency of the nomogram. The calibration curves for 1, 3, and 5 years were almost diagonal, indicating that the nomogram was reliable (Figures 5D–F). These results suggested that the risk score could not only be used as an independent prognostic factor but could also be used to predict the survival probability and death risk of ccRCC patients.

### Functional enrichment analysis of the nine prognosis-related genes

**FIGURE 6:**
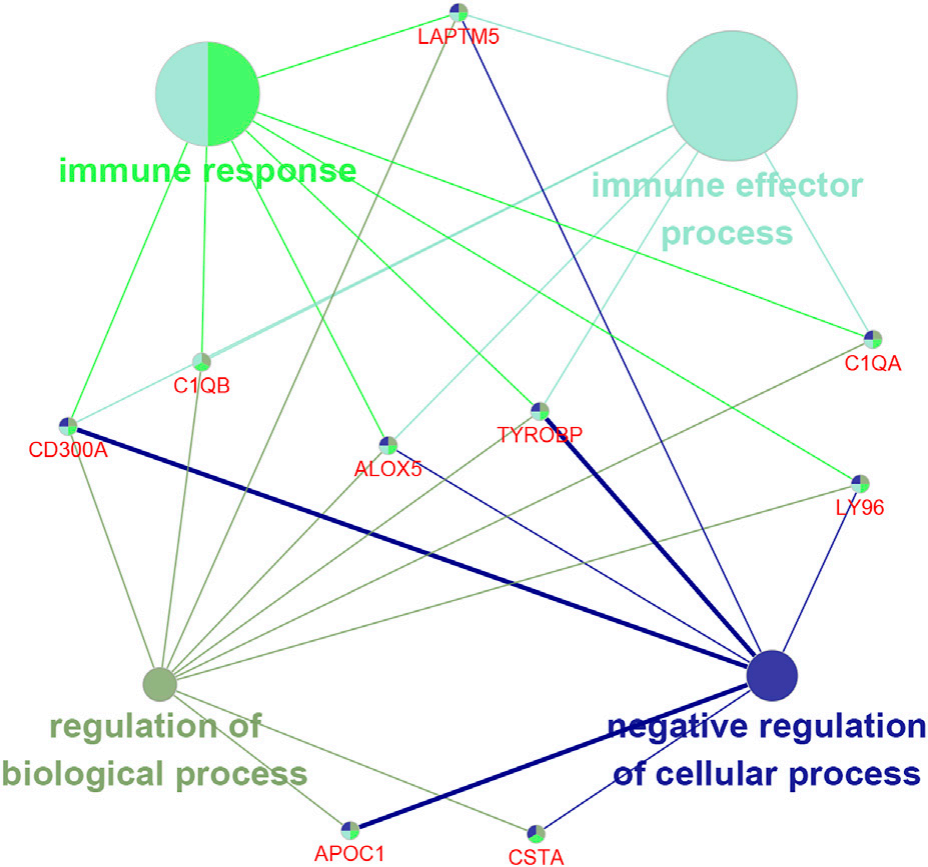
Functional enrichment analysis of the nine prognosis-related genes.

We used the ClueGO plug-in of Cytoscape software to analyze the functional relationship of the nine prognosis-related genes. The results showed that seven of the nine genes were enriched in the immune response, among which five genes were enriched in the immune effector process (Figure 6). This suggested that these genes might be involved in regulating the immune system.

### The relationship between risk score and immunotherapy

**FIGURE 7:**
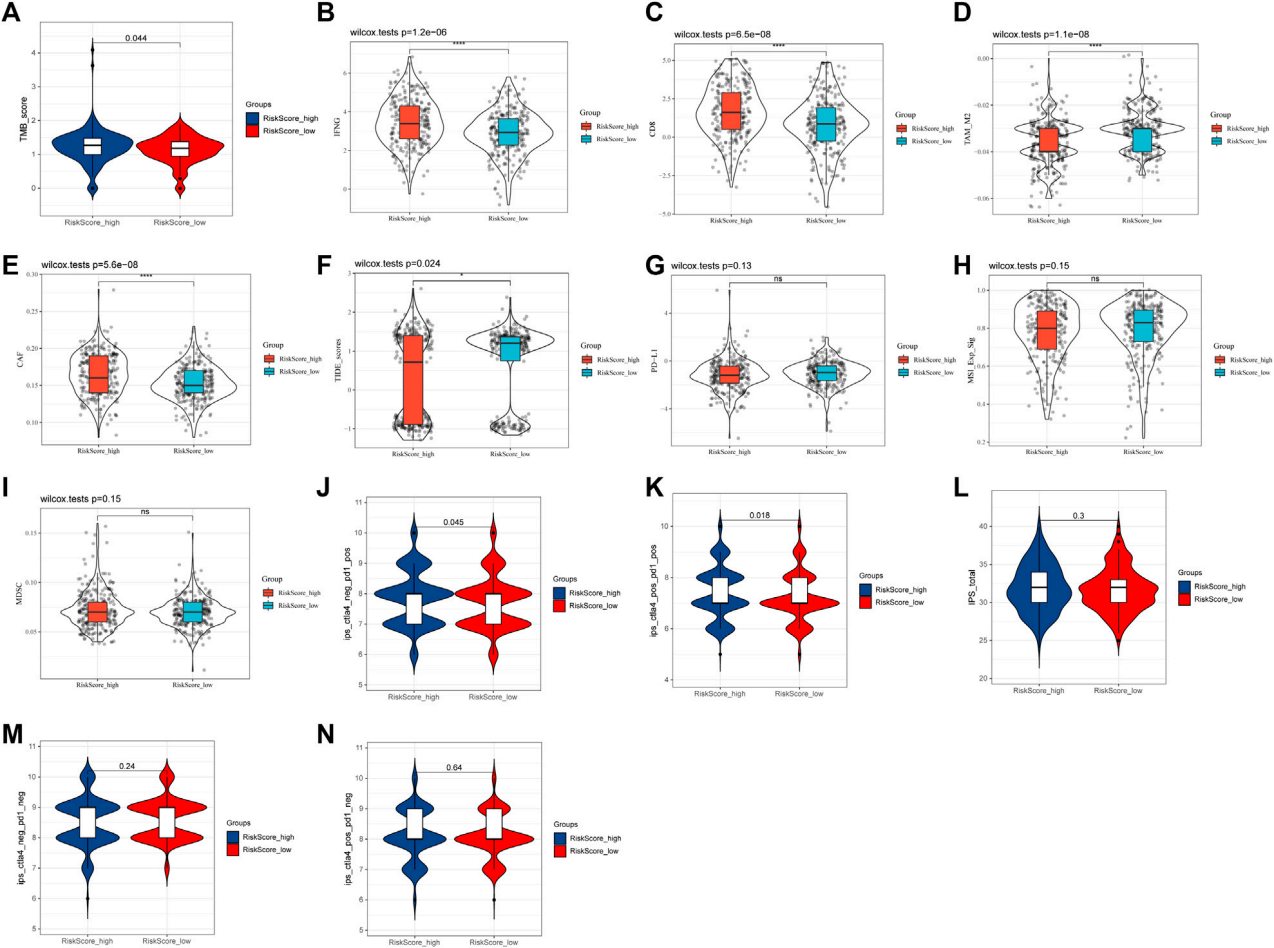
Relationship between the risk score and immunotherapy. **(A)** TMB score. **(B)** IFNG. **(C)** CD8. **(D)** TAM_M2. **(E)** CAF. **(F)** TIDE score. **(G)** PD-L1. **(H)** MSI_Exp_Sig. **(I)** MDSC. **(J)** IPS score with CTLA4_neg_PD1_pos. **(K)** IPS score with CTLA4_pos_PD1_pos. **(L)** Total IPS score. **(M)** IPS score with CTLA4_neg_PD1_neg. **(N)** IPS score with CTLA4_pos_PD1_neg.

According to the previous functional enrichment analysis, these genes may be involved in immune regulation. Thus, we first explored the relationship between the risk score and immunotherapy and found that the response ratio to immune checkpoint blockers (ICBs) in the high-risk group was significantly higher than that in the low-risk group (49.25% vs. 24.72%, χ^2^ = 34.409, *p* ≤ 0.001, Table 2). Similarly, the TMB score (*p* = 0.044, Figure 7A), IFNG (*p* = 1.2e-06, Figure 7B), CD8 (*p* = 6.5e-08, Figure 7C), TAM_M2 (*p* = 1.1e-08, Figure 7D), and CAF (*p* = 5.6e-08, Figure 7E) in the high-risk group were significantly higher than those in the low-risk group. The TIDE score in the high-risk group was significantly lower than that in the low-risk group (*p* = 0.024, Figure 7F). PD-L1 (*p* = 0.13, Figure 7G), MSI_Exp_Sig (*p* = 0.15, Figure 7H), and MDSCs (*p* = 0.15, Figure 7I) were not significantly different between the two groups. These results suggested that patients in the high-risk group might be more likely to benefit from immunotherapy.

**TABLE 2:** Response ratio to immune checkpoint blockers.

	High-risk group		Low-risk group		χ2	*p*
	n	%	n	%		
Total	266		267		34.409	≤0.001
R	131	49.25	66	24.72		
NR	135	50.75	201	75.28		

Note: R: response. NR: non-response.

In addition, the IPS score also showed that IPS scores with PD1_pos (both CTLA4_neg and CTLA4_pos) in the high-risk group were all significantly higher than those in the low-risk group (CTLA4_neg_PD1_pos: 7.84 vs. 7.70, *p* = 0.045, Figure 7J; CTLA4_pos_PD1_pos: 7.42 vs. 7.23, *p* = 0.018, Figure 7K). However, the total IPS score (*p* = 0.3, Figure 7L) and IPS scores with PD1_neg [both CTLA4_neg (*p* = 0.24, Figure 7M) and CTLA4_pos (*p* = 0.64, Figure 7N)] were not significantly different between the two groups. These results further suggested that the risk score, especially in the high-risk group, might be associated with PD-1-related immunotherapy.

### Exploration of the mechanism of action of the nine prognosis-related genes

**FIGURE 8:**
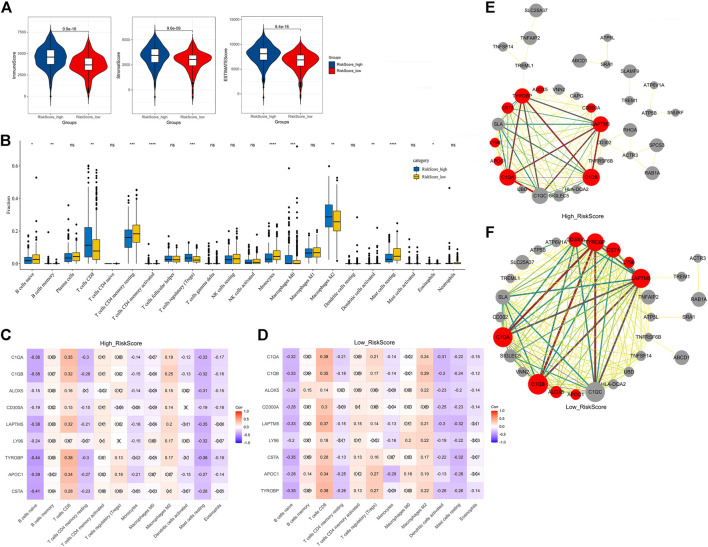
Relationship between the nine prognosis-related genes and immunity. **(A)** Immune score, stromal score, and ESTIMATE score. **(B)** Fractions of 22 immune cells in the high- and low-risk groups. **(C)** Correlation heatmap between the nine prognosis-related genes and significant immune cells in the high-risk group. **(D)** Correlation heatmap between the nine prognosis-related genes and significant immune cells in the low-risk group. **(E)** Correlation network between the nine prognosis-related genes and markers of activated dendritic cells in the high-risk group. **(F)** Correlation network between the nine prognosis-related genes and markers of activated dendritic cells in the low-risk group. × means not significant **(C–D)**. Red represents the nine prognosis-related genes, gray represents the markers with activated dendritic cells, and the thickness of the lines represents the strength of the correlation **(E–F)**.

To further explore and illustrate the mechanism of the nine prognosis-related genes in immune regulation, we first analyzed the relationship between these genes and the immune microenvironment. This analysis indicated that the immune score (*p* = 9.9e-16), stromal score (*p* = 9.6e-09), and estimated score (*p* = 8.4e-16) of the high-risk group were significantly increased compared with those of the low-risk group (Figure 8A). The results indicated that the nine prognosis-related genes were involved in regulating the immune microenvironment.

To further explore the relationship between these genes and immune cells, we first used the CIBERSORT algorithm to analyze the fractions of 22 immune cells in the high- and low-risk groups (Figure 8B). Excluding the immune cells without a significant difference between the two groups, we then calculated the Pearson correlation between the nine prognosis-related genes and the infiltrated immune cells in the high-risk group and low-risk group. The results indicated that in the high-risk group, the activated dendritic cells were only significantly negatively correlated with three genes (Figure 8C), while in the low-risk group, the activated dendritic cells were significantly negatively correlated with all nine genes (Figure 8D). This suggested that a reduction of activated dendritic cells was observed in the low-risk group.

To further analyze the correlation of the nine prognosis-related genes and dendritic cells, we downloaded the molecular markers of the dendritic cells and analyzed the correlations between the expression patterns of the markers and prognosis-related genes in the high-risk group and low-risk group. Consistent with the aforementioned results, there were more markers closely related to the nine genes expressed in the low-risk group than in the high-risk group (Figures 8E,F, Supplementary Tables S4, S5). These results further suggested that the nine prognosis-related genes seemed to be closely negatively related to activated dendritic cells in the low-risk group than in the high-risk group.

### Validation of the relationship between the prognosis-related genes and immunotherapy

**FIGURE 9:**
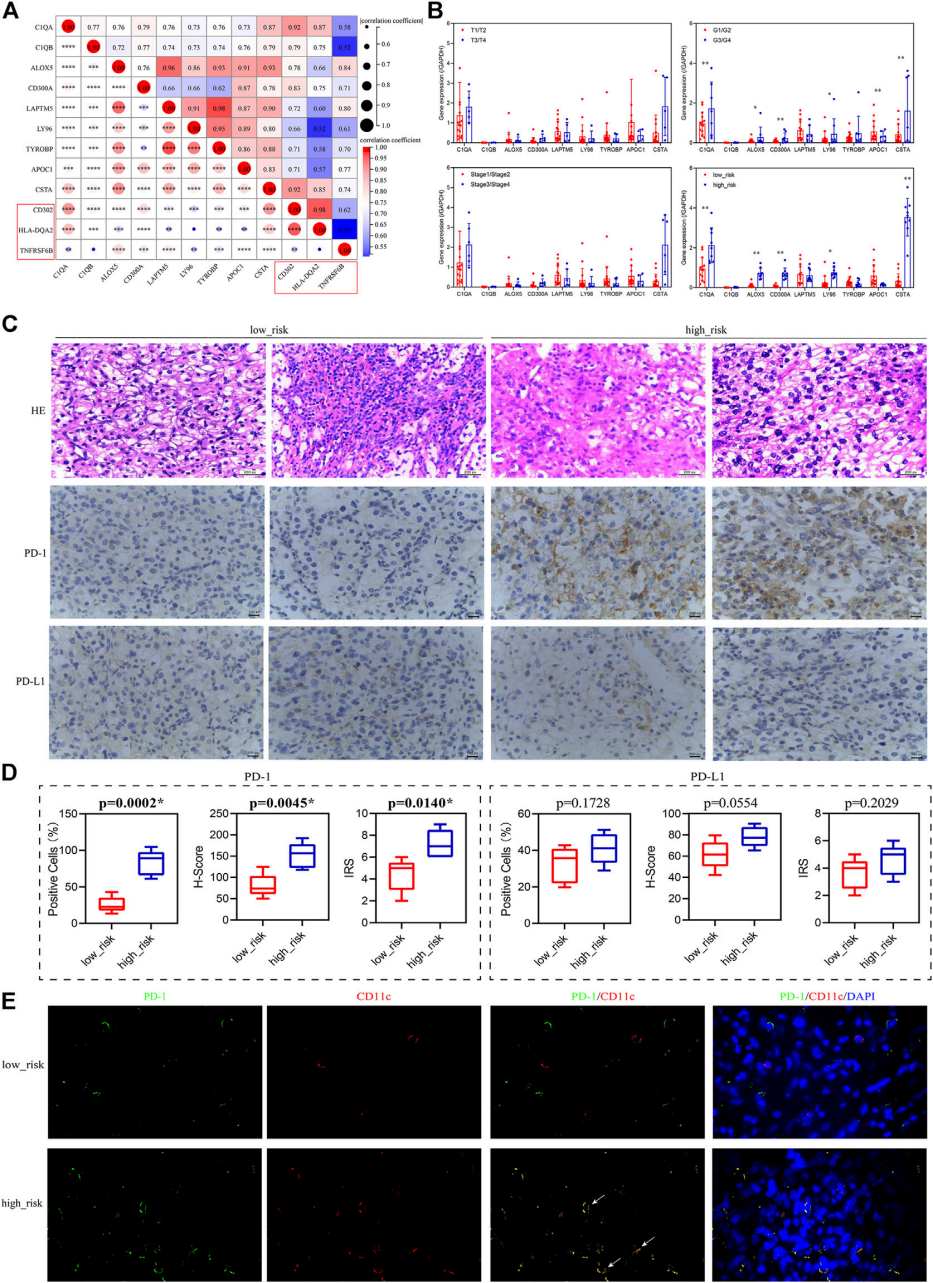
Validation of the relationship between the prognosis-related genes and immunotherapy. **(A)** Correlation of the prognosis-related genes and the activated dendritic cell markers (red box). **(B)** Expression levels of the prognosis-related genes in patients grouped with different clinical traits. T1–T4 represents T stage, which refers to the size and extent of the primary tumor, and is represented by T1–T4 in turn. G1–G4 represents clinicopathological grades, and the larger the number, the lower the degree of tumor differentiation, and the higher the degree of malignancy. Stage 1–Stage 4 represents clinical staging. **(C)** HE staining and immunohistochemistry (PD-1/PD-L1) of low-risk and high-risk patients, 400×. **(D)** Percentage of positive cells, H-Score, and IRS for immunohistochemical analysis of PD-1/PD-L1. **(E)** Immunofluorescence of PD-1 (green) and CD11c (red) in low-risk and high-risk patients, 1,000×, white arrows indicate red and green overlap.

To verify the relationship between the prognosis-related genes and immunotherapy, we collected tissue paraffin blocks and the clinical data of 22 ccRCC patients from Shaanxi Provincial People’s Hospital between January 2017 and January 2019 to identify the expression of the prognosis-related genes and activated dendritic cell markers and verify the correlations between them. We found that the prognosis-related genes were significantly correlated with the three markers (Figure 9A), further suggesting that the prognosis-related genes were associated with activated dendritic cells. The expression of the prognosis-related genes was significantly different between G1/G2 patients and G3/G4 patients (Figure 9B), which is consistent with the aforementioned results of the multivariate Cox regression analysis. However, their expression was not significantly different between T1/T2 patients and T3/T4 patients or between Stage 1/2 patients and Stage 3/4 patients (Figure 9B), which might be related to the small sample size. Furthermore, 22 ccRCC patients were divided into the low-risk group and high-risk group according to the median of all patients’ risk score calculated by the expression of nine prognosis-related genes and survival data. The follow-up deadline was January 2022, and the median follow-up time was 48 months. The expression of the prognosis-related genes was significantly different between the low-risk group and high-risk group (Figure 9B).

Since the prognostic-related genes were closely related to immunotherapy, we selected five low-risk patients and five high-risk patients from the 22 ccRCC patients to analyze their positive staining for PD-1 and PD-L1 (Figure 9C). The results showed that the percentage of positive cells, H-Score, and IRS for PD-1 in the high-risk group were significantly higher than those in the low-risk group, but for PD-L1, they had no significant difference between the high-risk group and low-risk group (Figure 9D), suggesting that PD-1 positivity might be correlated with the prognosis-related genes. Furthermore, to explore the expression of PD-1 in dendritic cells in tumor sections from ccRCC patients, we used the CD11c antibody (red) to label dendritic cells (Kvale et al., 2006) and performed immunofluorescence staining analysis with PD-1 antibody (green). The results indicated that the localization of PD-1 protein overlapped with the CD11c protein in the high-risk group, but not in the low-risk group (Figure 9E), suggesting that dendritic cells might express or bind PD-1 in the high-risk group.

## Discussion

CcRCC is a malignant tumor, and it still lacks specific diagnostic markers and therapeutic targets. To solve this problem, we analyzed the transcriptome and clinical data of ccRCC from the TCGA database to screen prognostic genes and verify their roles in the prognosis and immunotherapy of ccRCC. It was found that nine genes (TYROBP, APOC1, CSTA, LY96, LAPTM5, CD300A, ALOX5, C1QA, and C1QB) not only had high prognostic value but also the risk score constructed by them was significantly correlated with immunotherapy-related indicators such as TIDE score, TMB, PD-1, and ICB response rate.

The risk score constructed by the nine prognostic-related genes had an independent prognostic value and was associated with poor prognosis of ccRCC. It could predict the 1-/3-/5-year survival probability of patients, suggesting that these genes play important roles in the occurrence, progression, and metastasis of various tumors. Furthermore, the multivariate Cox regression analysis showed that the risk score had a potential prognostic value and a role in pathological grade and stage. Some previous studies have also supported this hypothesis and have reported the prognostic value of individual genes in a variety of tumors. For example, APOC1 is closely related to poor prognosis in ccRCC patients, as well as age, clinical stage, and pathological grade (Xiao and Xu, 2021). C1QA and C1QB might be potential prognostic factors and indices of tumor microenvironment remodeling in osteosarcoma (Chen et al., 2021). Elevated TYROBP expression predicted poor survival in patients with low-grade glioma (Lu et al., 2021). In contrast to the aforementioned studies, we combined multiple genes to construct a risk score model and nomogram and found that they had a good prognostic value. Therefore, we hope that the score derived from these nine prognostic-related genes can provide a predictive value for prognosis of clinical ccRCC patients.

PD-1/PD-L1, TMB, and MSI/MMR are independent and synergistic in immunotherapy (Vanderwalde et al., 2018). The higher the level of each index above its cutoff, the higher the probability the tumor will respond to immunotherapy, except for MMR (Zhu et al., 2018; Tucker and Rini, 2020). The lack of MMR protein expression predicts a benefit from immunotherapy (Chen et al., 2018). The TIDE score can be used to evaluate the response rate of patients to ICB therapy; the higher the TIDE score, the lower the ICB response rate (Jiang et al., 2018). These findings are consistent with our research results. The patients in the high-risk group had lower TIDE scores and higher TMB levels, PD-1 levels, and ICB response rates. The TIDE score predicts outcomes in melanoma patients who receive first-line anti-PD1 or anti-CTLA4 therapy by mimicking two escape mechanisms of tumors (T cell dysfunction signature and T cell exclusion signature) more accurately than other biomarkers, such as PD-L1 levels and mutation load (Jiang et al., 2018). However, KIRC has a stronger propensity for T cell dysfunction signature, so the relationship between the TIDE score and risk signature in this article requires a large number of samples to continue to be validated. In addition, the levels of MSI, PD-L1, and CTLA4 showed no significant differences between the high- and low-risk groups, which may be related to tumor heterogeneity and the relative independence of each indicator. Among all solid tumors, the proportion of high TMB, PD-L1, and MSI is only 73/11348 (Vanderwalde et al., 2018). In addition, we also noticed that the level of PD-1 in the high-risk group was higher than that in the low-risk group, while there was no significant difference in the level of PD-L1. The reason for this may be that, under normal circumstances, PD-1 in combination with the ligand PD-L1 downregulates the immune system’s response to its own cells and promotes self-tolerance. However, in tumors and during inflammation, upregulation of PD-1 and/or PD-L1 causes immune cells to escape the autoimmune response (Sun et al., 2018). However, that did not affect our conclusions. The risk score constructed from the nine prognostic-related genes could effectively predict the response to immunotherapy, and it might be considered a new immunotherapy evaluation index for ccRCC.

The principle of immunotherapy mainly includes two aspects: one is to improve autoimmunity to eliminate tumor cells, such as the application of dendritic cell vaccines, and the other is to reduce the immunity of tumor cells and enhance the recognition of the autoimmune system to eliminate the immune escape of tumor cells, finally eliminating the tumor cells, such as the application of ICIs (Santos and Butterfield, 2018; Waldman et al., 2020). Our results with the correlation heatmap and PD-1 immunohistochemistry showed that the low-risk group was significantly negatively associated with the activated dendritic cells and had a lower PD-1 positive staining rate, suggesting that the activated dendritic cells were more lacking in the low-risk group, which indicated dendritic cell activation in the high-risk group. This was also confirmed by previous results. We detected a lower TMB in the low-risk group, indicating that it had fewer cancer cell neoantigens that would not drive its release and presentation to antigen-presenting cells, such as dendritic cells, so it could maintain the function of antigen-presenting cells and avoid tumor escape events (Chen and Mellman, 2013; Braun et al., 2021). In contrast, the high-risk group had higher tumor cell immune escape. At the same time, we observed a higher PD-1 positive staining rate and a combination of dendritic cells and PD-1 in the high-risk group, suggesting that poor prognosis of patients in the high-risk group may be due to the binding of dendritic cells to PD-1, resulting in an inhibitory immune response (Yao et al., 2009). Currently, the antibodies targeting the PD-1 and CTLA4 immune checkpoints have been successful in renal cell carcinoma (Bellone and Elia, 2017; Braun et al., 2021). Emerging data suggested that patients whose tumors overexpressed PD-1/PD-L1 by IHC had improved clinical outcomes with anti-PD-1-directed therapy (Patel and Kurzrock, 2015). Therefore, patients in the high-risk group might have a better clinical outcome if they receive anti-PD-1 immunotherapy. We deduced that if the high-risk group is treated with ICIs, it would be easier to activate dendritic cells than in the low-risk group and reduce the immune escape of the tumor cells, ultimately eliminating the tumor cells to achieve immunotherapy. Taken together, the patients in the high-risk group were more likely to benefit from immunotherapy.

However, this study also has certain limitations, such as a small sample size and a lack of clinical data on immunotherapy for verifying the relationship between the risk score and immunotherapy. In future studies, we will expand the sample size and focus on the relationship between the nine prognostic-related genes/risk score and dendritic cells, immunotherapy, and immune escape, applying both clinical data and laboratory methods to screen for immunotherapy targets more accurately.

In conclusion, the risk score constructed by the nine prognostic-related genes could predict the survival rate of ccRCC patients. Moreover, the patients in the high-risk group were more likely to benefit from immunotherapy, and the mechanism might be related to the release of dendritic cell-mediated immunosuppression. Our study demonstrates the guiding value of this risk score for immunotherapy and enriches the known immunotherapy predictors, aiming to provide new predictive methods and therapeutic targets for immunotherapy of tumors.

## Data Availability

The datasets presented in this study can be found in online repositories. The names of the repository/repositories and accession number(s) can be found in the article/Supplementary Material.

## Abbreviations

TMB, tumor mutational burden; TIDE, tumor immune dysfunction and exclusion; PD-L1, programmed cell death-ligand 1; MSI, microsatellite instability; IFNG, interferon gamma; TAMs, tumor-associated macrophages; CAFs, carcinoma-associated fibroblasts; MDSCs, myeloid-derived suppressor cells; CTLA4, cytotoxic T - lymphocyte antigen 4; PD-1, programmed cell death 1.
